# Efficient *k*-Winner-Take-All Competitive Learning Hardware Architecture for On-Chip Learning

**DOI:** 10.3390/s120911661

**Published:** 2012-08-27

**Authors:** Chien-Min Ou, Hui-Ya Li, Wen-Jyi Hwang

**Affiliations:** 1 Department of Electronic Engineering, Ching Yun University, Jhongli 320, Taiwan; 2 Department of Computer Science and Information Engineering, National Taiwan Normal University, Taipei 116, Taiwan

**Keywords:** reconfigurable computing, system on programmable chip, FPGA, competitive learning, *k*-winners-take-all

## Abstract

A novel *k*-winners-take-all (*k*-WTA) competitive learning (CL) hardware architecture is presented for on-chip learning in this paper. The architecture is based on an efficient pipeline allowing *k*-WTA competition processes associated with different training vectors to be performed concurrently. The pipeline architecture employs a novel codeword swapping scheme so that neurons failing the competition for a training vector are immediately available for the competitions for the subsequent training vectors. The architecture is implemented by the field programmable gate array (FPGA). It is used as a hardware accelerator in a system on programmable chip (SOPC) for realtime on-chip learning. Experimental results show that the SOPC has significantly lower training time than that of other *k*-WTA CL counterparts operating with or without hardware support.

## 1. Introduction

The *k*-winners-take-all (*k*WTA) operation is a generalization of the winner-take-all (WTA) operation. The *k*WTA operation performs a selection of the *k* competitors whose activations are larger than the remaining input signals. It has important applications in machine learning [1], neural networks [2], image processing [3], mobile robot navigation [4] and others [5–8]. One drawback of *k*-WTA operations is the high computational complexities when number of input signals is large. Therefore, for realtime *k*WTA-based applications, hardware implementation of *k*WTA is usually desirable. There have been many attempts to design hardware circuits for *k*WTA operations. Nevertheless, many architectures are designed by analog circuits [2,9] with constraints on input signals. Because the input signals are generally not known beforehand, the circuits may not produce correct results when the constraints are not met. Although some circuits have lifted the constraints [10], overhead for the implementation of analog/digital converter is still required when the circuits are used for digital applications. In addition, some digital circuits [11–13] can only detect winners for one input set at a time. The throughput of the circuits may be further enhanced by allowing different winner detection operations sharing the same circuit at the same time.

Similar to the WTA/*k*WTA circuits, the winner detection is an important task in the winner-take-most (WTM) and neural gas hardware architectures. However, some hardware architectures [14,15] for WTM and neural gas networks are still based on analog circuits. Similar to the digital circuits [11–13] for WTA, the digital architecture for WTM [16] performs winner detection only for one input set at a time. The neural gas architecture [17] separates the winner detection operation into two phases: the distance computation, and sorting. These two phases are performed in an overlapping fashion. However, the sorting phase is implemented by software. Therefore, the architecture [17] may only attains limited throughput.

This paper presents a novel *k*WTA hardware architecture performing the concurrent winner detection operations over different input sets. The proposed architecture is suitable for digital implementation, and imposes no constraints on the input signals. To demonstrate the effectiveness of the proposed architecture, a novel *k*WTA-based competitive learning (CL) circuit using the proposed architecture is built. The CL algorithm has been widely used as an effective clustering technique [18,19] for sensor devices [20,21], wireless sensor networks [22,23], data approximation [24], data categorization [25] and information extraction [26]. In a CL network, the neurons compete among themselves to be activated or fired. The weight vector associated with each neuron corresponds to the center of its receptive field in the input feature space.

The CL with *k*WTA activation can be separated into two processes: competition and updating. Given an input training vector, the competition process finds the *k* best matching weight vectors to the input vector. The updating process then updates the *k* winners. Although existing *k*WTA architectures can be used for expediting the CL, these architectures only find the *k* winners for one input set at a time. The set of distance associated with each weight vector is used as the input set for the *k*WTA circuits. Different input training vectors result in different input sets. Consequently, the competition process can only be performed for one input training vector at a time. These architectures may then provide only moderate acceleration.

The proposed architecture accelerates the training process by performing the competitions associated with different input training vectors in parallel. Different input sets share the same *k*WTA circuit by the employment of pipeline with codeword swapping. In the proposed architecture, each training vector is allowed to carry its current *k* best matching neurons as it traverses through the pipeline. By incorporating the codeword swapping at each stage of the pipeline, neurons failing the competition for a training vector are then immediately available for the competitions for the subsequent training vectors. When a training vector reaches the final stage of the pipeline, a hardware-based neuron updating process is activated. The process involves the computation of learning rate and new weight vector for the winning neuron. To accelerate the process, a lookup table based circuit for finite precision division is utilized. It is able to reduce the computational time and lower the area cost at the expense of slight degradation in training process. The combination of codeword swapping scheme for neuron competition and lookup table based divider for neuron updating effectively expedites the CL training process.

The proposed architecture has a number of advantages. The first advantage of the architecture is the high throughput. Different training vectors are able to share the pipeline for the *k*WTA operations. The number of training vectors sharing the pipeline increases with the number of neurons. Hence, the throughput enhancement becomes very prominent as the number of neurons becomes large. An additional advantage is the low area cost. Only the comparators and multiplexers are involved in the *k*WTA operation. Although the tree architecture [27] is also beneficial for enhancing the throughput for the winner detection, it may need extra hardware resources. This is because the circuit needs additional intermediate nodes to build a search tree accelerating winner detection process. Each intermediate node may contain a distance computation unit, resulting in large area costs for hardware implementations.

In addition to the high throughput and low area cost, the proposed architecture can move the best *k* matching vectors to an input training vector to the final *k* stages of the pipeline, because of the employment of the codeword swapping scheme. As the number of neurons become large, after the *k* best matching neurons are identified, efficient retrieval of the these *k* winners may be complicated. By moving the winners to the final stages of the pipeline, the post-*k*WTA operations such as the updating process in the CL can operate directly on the final *k* stages of the pipeline.

To physically measure the performance, the proposed architecture has been implemented on field programmable gate array (FPGA) devices [28,29] so that it can operate in conjunction with a softcore CPU [30]. Using the reconfigurable hardware, we are then able to construct a system on programmable chip (SOPC) system for the CL clustering. In this paper, comparisons with the existing software and hardware implementations are made. Experimental results show that the proposed architecture attains a high speedup over its software counterpart for the *k*WTA CL training. It also has a lower latency over existing hardware architectures. Our design therefore is an effective alternative for the applications where realtime *k*WTA operations and/or CL training are desired.

## 2. The Proposed Architecture

### 2.1. The CL Algorithm with k-WTA Activation

Consider a CL network with *N* neurons. Let **y***_i_, i* = 1, …, *N*, be the weight vectors of the network. In the CL algorithm with *k*-WTA activation, given a training vector **x**, the squared distance *D*(**x, y***_i_*) between **x** and **y***^i^* is computed. The dimension of input vectors and weight vectors is 2*^n^* × 2*^n^*. Any weight vector **y**_i__*_ belonging to 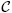
(**x**) will be updated, where 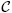
(**x**) is the set of the *k* best matching weight vectors to **x**. The updated y_i__*_ is then given as:

(1) $${\text{y}}_{i*}\leftarrow {\text{y}}_{i*}+\eta_{i*}(\text{x}-{\text{y}}_{i*})$$

where η_i__*_ is the learning rate of the **y**_i__*_. Discovering the vector 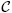
(**x**) requires an exhaustive search over *N* vectors. When *N* and/or *n* is large, the computational complexity of CL algorithm is very high.

### 2.2. The Architecture Overview

**Figure 1.**
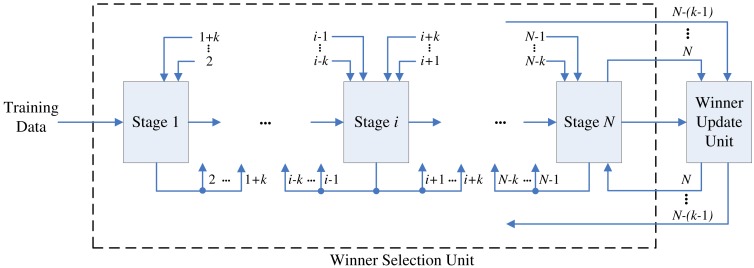
The proposed CL architecture.

The proposed architecture is a (*N* + 1)-stage pipeline for a CL network with *N* neurons, as shown in Figure 1. The architecture can be divided into two units: winner selection unit and winner update unit. The winner selection unit includes *N* stages, where each stage contains one neuron in the CL network. The goal of winner selection unit is to find the set of *k* best matching vectors to **x**. The winner selection unit is therefore a *k*WTA circuit.

**Figure 2.**
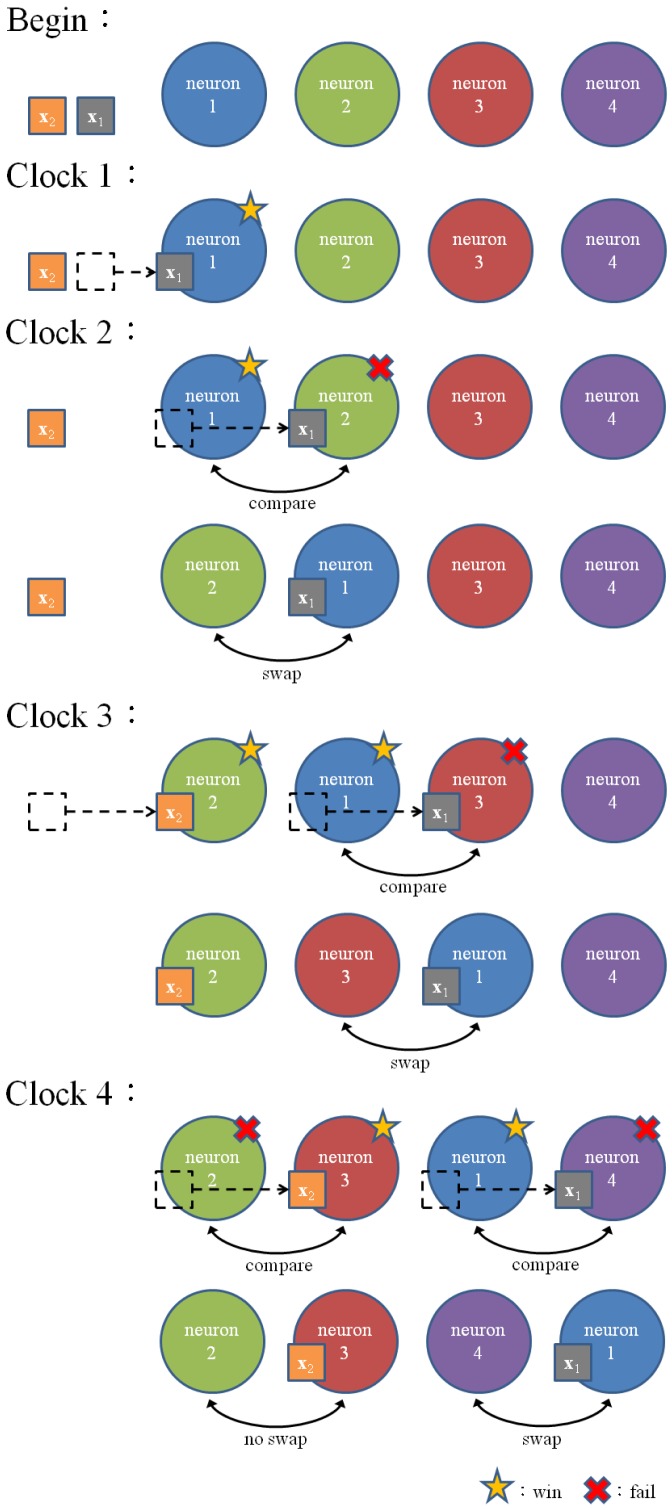
An example of codeword swapping scheme for *N* = 4 and *k* = 1.

To allow multiple training vectors concurrently sharing the winner selection unit for the *k*WTA operation, a codeword swapping operation is adopted by the pipeline. By the employment of the codeword swapping circuit, the *k* best matching neurons can be traversed through the pipeline with the training vector. Figure 2 shows an example of codeword swapping scheme. For the sake of simplicity, there are only 4 neurons in the network (*i.e., N* = 4) with *k* = 1. Assume that the weight vector associated with the first neuron is closest to the current training vector **x** (i.e., j* = 1). As shown in Figure 2, when a training vector enters each stage, the codeword swapping circuit will be activated for that stage so that the best matching neuron can also be traversed through the pipeline with the training vector.

**Figure 3.**
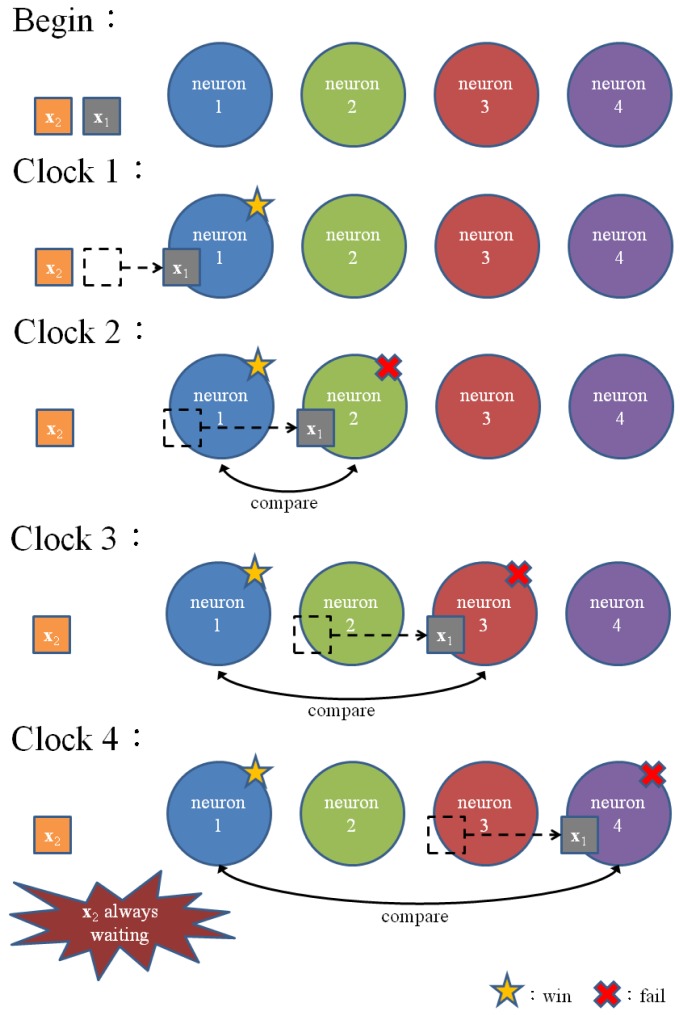
An example of pipeline without codeword swapping scheme.

Without the codeword swapping scheme, the best matching neuron will always stay at the first stage, as shown in Figure 3. The subsequent training vectors are not able to enter the pipeline until the best matching neuron is updated in accordance with Equation (1). Note that the neuron updating process will be activated only when the competition at the final stage of the pipeline for the current training vector is completed. Therefore, in the case without the codeword swapping scheme, the pipeline may process only one training vector at a time. On the contrary, when the codeword swapping is employed, the neurons failing the competition will be moved forward. They will then be available for the competition for the subsequent training vectors. The proposed pipeline therefore will process the *k*WTA for multiple training vectors concurrently.

**Figure 4.**
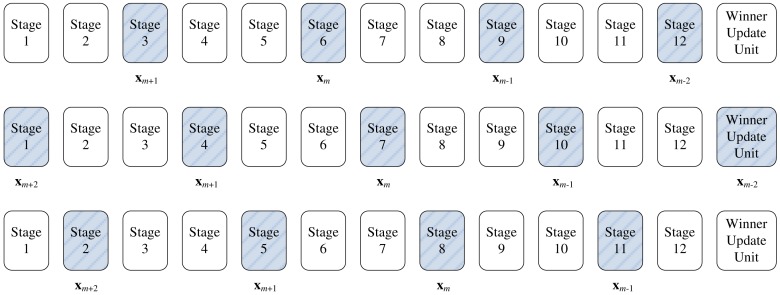
Snap shots of the proposed pipeline architecture with *N* = 12 and *k* = 2, where shaded stages are the stages occupied by a training vector, and unshaded stages are the vacant stages.

To implement the codeword swapping scheme, successive training vectors are *k* stages apart in the pipeline. The first *k* stages before a training vector **x** in the pipeline store the current set of *k* winners 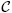
(**x**) associated with that training vector. Figure 4 gives a snapshot of the proposed architecture for *k* = 2. It can be observed from the figure that the pipeline allows up to ⌈(*N* +1)/(*k* + 1)⌉ competitions.

**Figure 5.**
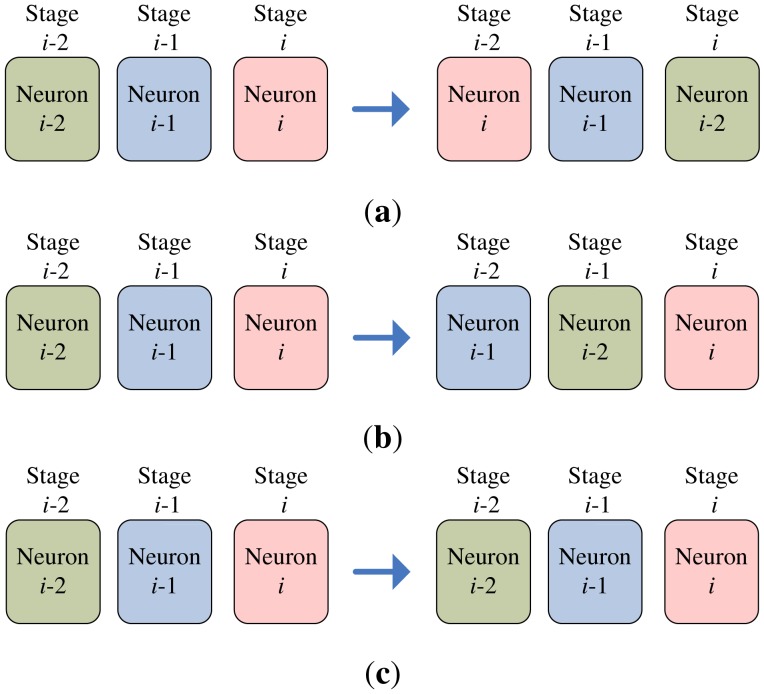
The codeword swapping operations for *k* = 2: (**a**) Loser is at stage *i*, (**b**) Loser is at stage *i* − 1, (**c**) Loser is at stage *i* − 2.

The codeword swapping operations are further elaborated in Figure 5 for *k* = 2. Assume a training vector **x** is at stage *i*. The current two winners associated with the **x** then reside at stages *i* − 2 and *i* − 1, respectively. The three neurons at stages *i* − 2, *i* − 1 and *i* then compete for the **x**. The loser will be swapped with the neuron at stage *i* − 2. After the swapping operations, the neuron at stage *i* − 2 is available for the competition for the next input vector.

The swapping operations for any *k* > 0 when a training vector **x** is currently at stage *i* can be extended easily. In this case, the current set of *k* winners 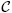
(**x**) are located from stage *i* − *k* to stage *i*. The *k* + 1 neurons (*i.e.*, the neurons at stages *i* − *k*, …, *i*) now competing for **x**. The worst matching neuron will then be swapped with the neuron at stage *i* − *k*.

**Figure 6.**
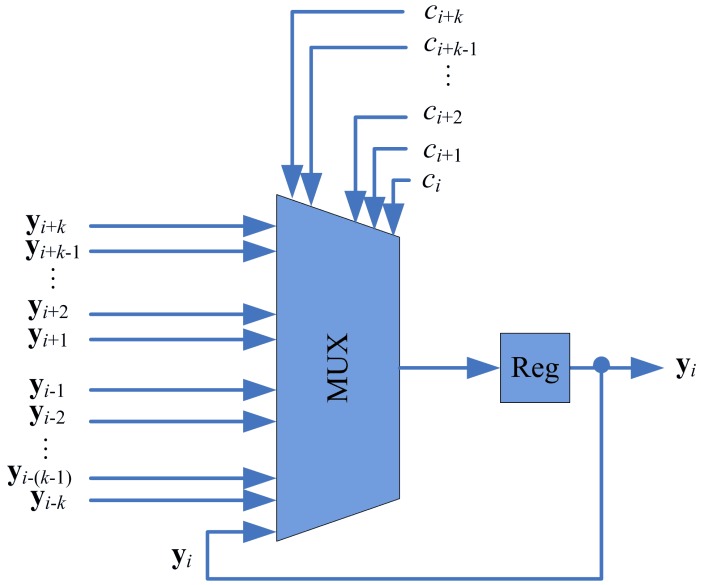
The architecture of the swap unit.

To implement the swapping operation, each stage of the proposed pipeline architecture contains a swap unit for the implementation of swapping operations. Figure 6 shows the architecture of the swap unit at each stage *i*, which consists of a register and a multiplexer. The register contains **y***_i_*, the current weight vector associated with stage *i*. The multiplexer consists of 2*k* + 1 inputs: **y***_i_*_−_*_k_*, …, **y***_i_*_+_*_k_*. The *k* + 1 control lines *c_i_*, …, *c_i_*_+_*_k_* determine the output of the multiplexer. The *c_i_* indicates the competition results at stage *i*. The *c_i_* takes the values in the set {0, 1, …, *k* + 1} such that

(2) $$c_{i}=\{\begin{array}{ll} 0 & \text{Stage}\;i\;\text{is vacant without training vector},\\ j+1 & \text{A training vector}\;\text{x}\;\text{is at}\;i,\text{and stage}\;i-j\;\text{loses competition} \end{array}$$

When **x** is at stage *i*, only stages *i, i* − 1, …, *i* − *k*, are involved in the competition. Therefore, 0 ≤ *j* ≤ *k*. Based on *c_i_* defined in Equation (2), the operation of the multiplixer can be designed. Table 1 shows the truth table of the multiplixer for *k* = 2. The truth table for can be easily extended for any *k* > 2.

**Figure 7.**
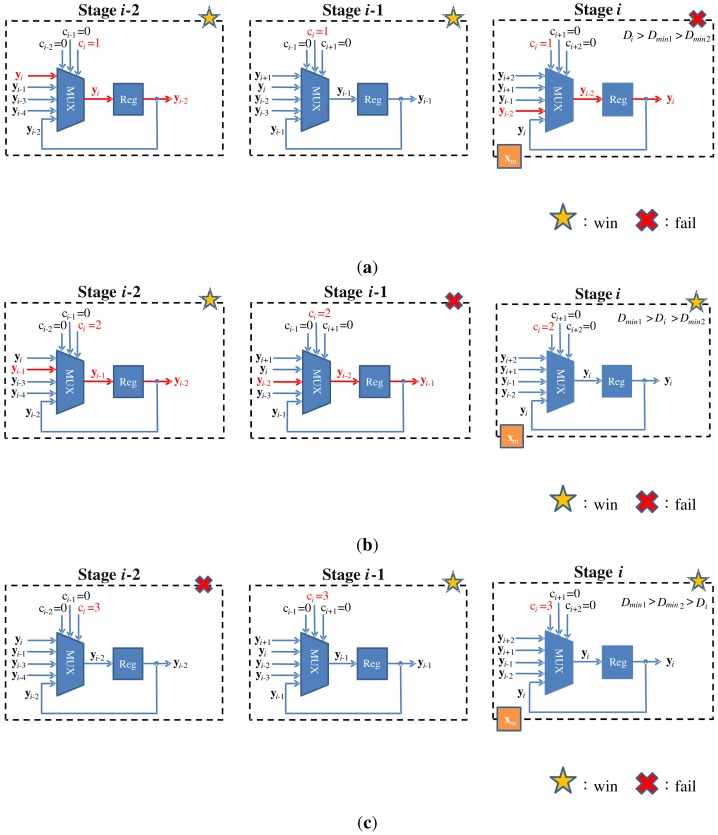
An example of operations of swap unit at stages *i, i* − 1 and *i* − 2 for *k* = 2. (**a**) Loser is at stage *i*, (**b**) Loser is at stage *i* − 1, (**c**) Loser is at stage *i* − 2.

An example of the operations of swap unit is shown in Figure 7 for *k* = 2. In this example, assume a training vector is at stage *i*. Because *k* = 2, only the swap units at stages *i, i* − 1 and *i* − 2 are considered. Moreover, because **x** is at stage *i*, the stages *i* − 2, *i* − 1, *i* + 1 and *i* + 2 are vacant without training vector. Based on Equation (2), *c_i_*_−2_ = *c_i_*_−1_ = *c_i_*_+1_ = *c_i_*_+2_ = 0. The value of *c_i_* will be 1, 2 or 3, dependent on the location of the neuron failing the competition. Figure 7 shows the swapping operation for each value of *c_i_*. It can be observed from Figure 7 that with simple multiplexers, the loser will always be moved forward to stage *i* − 2, while the two winners are moved backward to stages *i* − 1 and *i*. In this way, the loser is available to join the competition for subsequent training vectors.

### 2.3. The Architecture of the Winner Selection Unit

**Figure 8.**
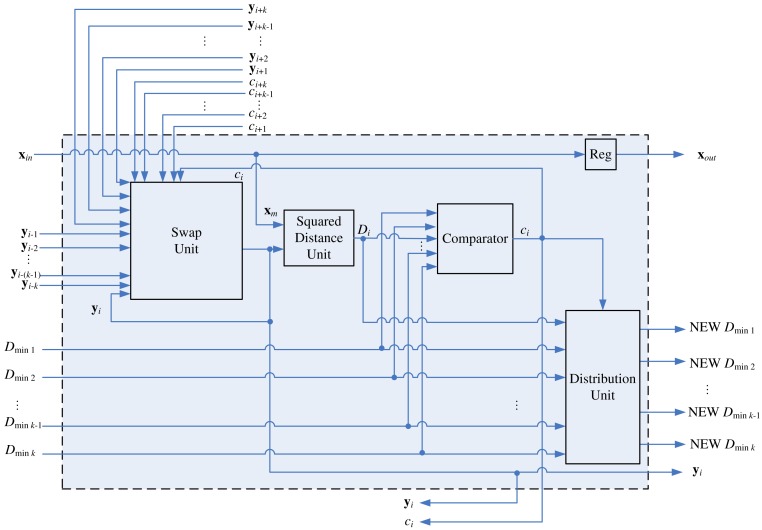
The architecture of the stage *i* of the pipeline, *k* < *i* ≤ *N* − *k*.

Figure 8 depicts the architecture of the stage *i* of the pipeline, *k* < *i* ≤ *N* − *k*, which consists of a swap unit, a squared distance unit, a comparator, and a distribution unit. Although the swap unit is the core part of the pipeline, other components are also necessary for determining the competition results *c_i_*.

The goal of the squared distance unit is to compute *D*(**x, y***_i_*), the distance between the training vector **x** and the weight vector at stage *i*, where *D*(**u, v**) is the squared distance between **u** and **v**. For sake of simplicity, we let

(3) $$D_{i}=D(\text{x},{\text{y}}_{i})$$

The comparator in the architecture is used for determining the competition result *c_i_*. As shown in Figure 8, in addition to *D_i_, D_min_j__*, the squared distance between **x** and **y***_i_*_−_*_j_, j* = 1, …, *k*, are the inputs to the comparator, where

(4) $$D_{{\text{min}}_{j}}=D(\text{x},{\text{y}}_{i-j})$$

**Figure 9.**
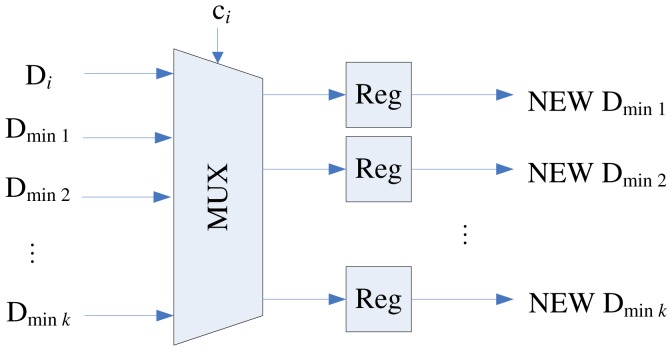
The architecture of the distribution unit.

Note that the current *D*_*min*_1__, …, *D_min_k__* are *not* necessarily in ascending or descending order. After *c_i_* at each stage *i* is computed, the swap unit will be activated for the codeword swapping operation. In addition, all the *D_min_j__, j* = 1, …, *k*, will be updated and stored in the distribution unit. The updating process should be consistent with the swapping process so that as **x** proceeds to the next stage (*i.e., i* ← *i* + 1), the new *D_min_j__* actually represents the squared distance between **x** and new **y***_i_*_−_*_j_*. The architecture of the distribution unit is shown in Figure 9, which contains a (*k* + 1) × *k* switch unit and registers. The switch unit has *k* + 1 inputs: *D_i_* and old *D*_*min*_1__, …, *D_min_k__*. Based on the *c_i_* value, the switch unit then remove one of the inputs, and re-shuffle the other *k* inputs to create the new *D*_*min*_1__, …, *D_min_k__*. Table 2 shows the operations of switch unit for *k* = 2 at stage *i*. The operations for larger *k* values can be easily extended by analogy.

**Figure 10.**
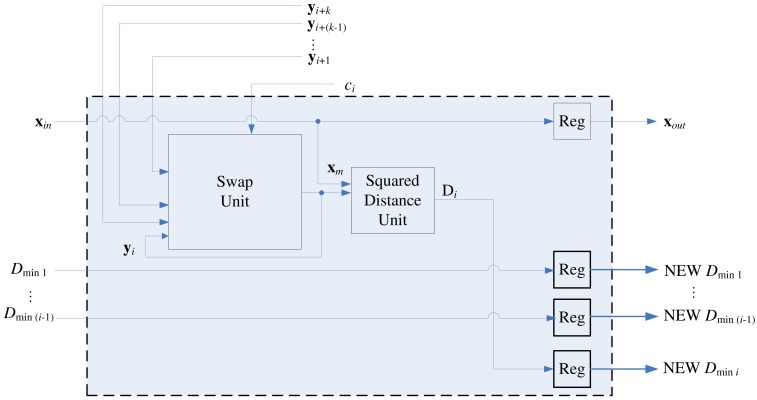
The architecture of stage *i* for 1 ≤ *i* ≤ *k*.

Figure 10 depicts the architecture of the stage *i* for 1 ≤ *i* ≤ *k*, which is the simplified version of the architecture shown in Figure 8. At the first *k* stages of the pipeline, because not all the vectors {**y***_i_*_−_*_k_*, …, **y***_i_*_−1_} in 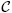
(**x**) are available as **x** enters these stages, no comparison to *D*_*min*_1__, …, *D_min_k__* is necessary. The comparator and distribution unit are removed, and the *c_i_* is always 0 at these stages.

**Figure 11.**
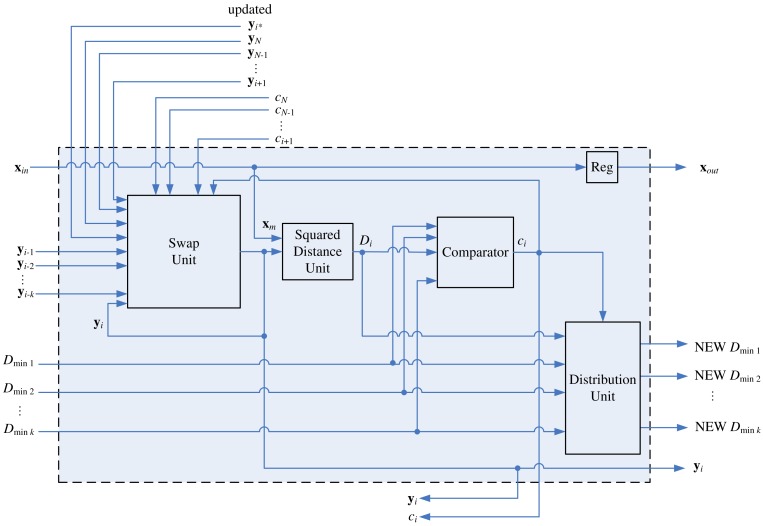
The architecture of stage *i* for *N* − *k* < *i* < *N*.

**Figure 12.**
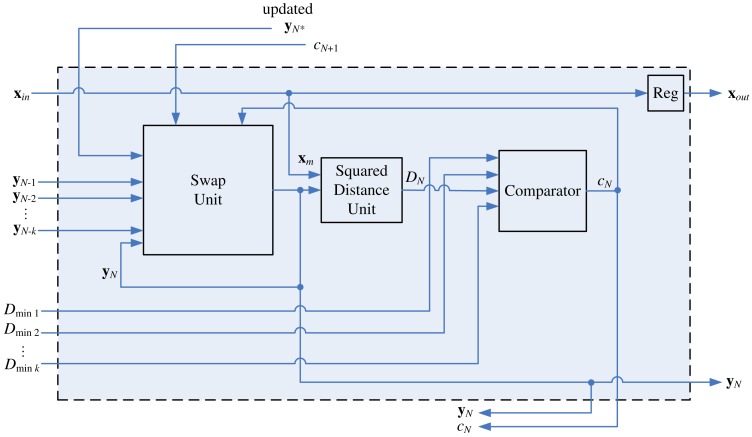
The architecture of stage *N*.

The architecture of stage *i* for *N* − *k* < *i* < *N* is depicted in Figure 11. All neurons at these stages will be delivered to the winner update unit for weight vector updating as the training vector **x** enters the winner update unit. In addition, updated neurons in 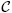
(**x**) do not stay at the winner update unit. They will be sent back to stages where they come from. Therefore, as shown in Figure 11, an updated neuron from stage *N* + 1 is also an input vector to the swap unit. The architecture of stage *N* is shown in Figure 12. In the architecture, it is not necessary to update and store new *D*_*min*_1__, …, *D_min_k__* because the stage *N* is the final stage of the winner selection unit. The neuron competition is no longer necessary for the subsequent operations.

### 2.4. The Architecture of the Winner Update Unit

**Figure 13.**
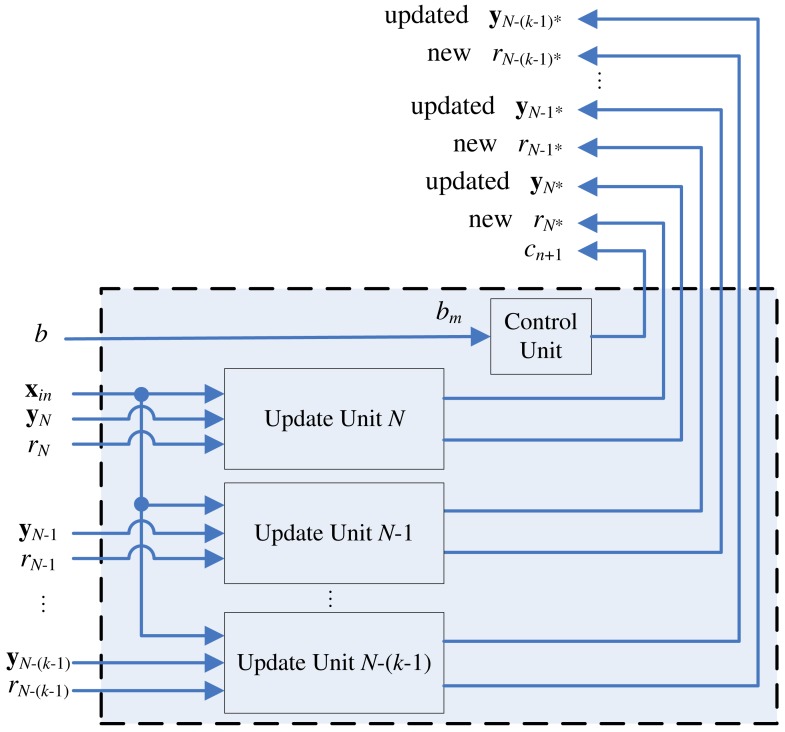
The architecture of the winner update unit.

Figure 13 shows the architecture of the winner update unit. As shown in the figure, there are *k* weight vector update modules in the architecture. These modules are responsible for updating weight vectors obtained from the final *k* stages of the winner selection unit, which are the actual *k* winners when the training vector **x** enters the winner update unit.

**Figure 14.**
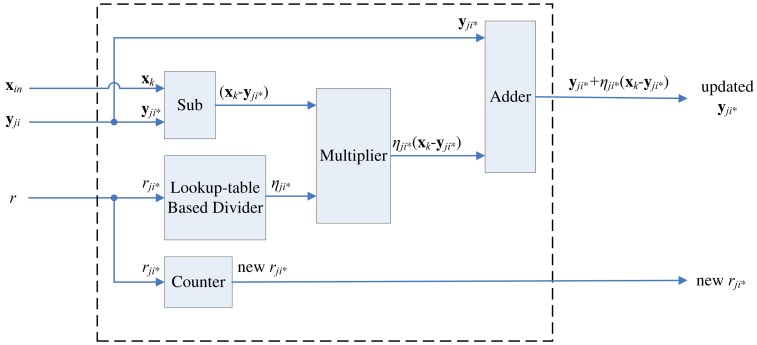
The architecture of the weight vector update module.

Let **y**_i__*_ ∈ *C*(**x**) be a real winner at the final *k* stages of the winner selection unit. Each update module computes the learning rate and updates new codeword for a **y**_i__*_, as shown in Figure 14. In the proposed architecture, the learning rate is given by

(5) $$\eta_{i*}=\frac{1}{4\times r_{i*}}$$

where r_i__*_ denotes current number of times the weight vector **y**_i__*_ wins the competition. The counter in the module is used for computing r_i__*_.

To compute the learning rate, each codeword **y***_i_* should be associated with its own *r_i_*. When **y***_i_*_−_*_j_* and **y***_i_* are decided to be swapped, *r_i_*_−_*_j_* and *r_i_* will be swapped as well. For the sake of brevity, the circuit for swapping *r_i_*_−_*_j_* and *r_j_* at each stage *i* is not shown in Figures 8, 10, 11 and 12.

After the actual winner has been identified at the final *k* stages, their r_i__*_ will be increased by 1 by the counter. The computation of learning rate involves division. In our design, a lookup table based divider is adopted for reducing the area complexity and accelerating the updating process.

### 2.5. The Proposed Architecture for On-Chip Learning

**Figure 15.**
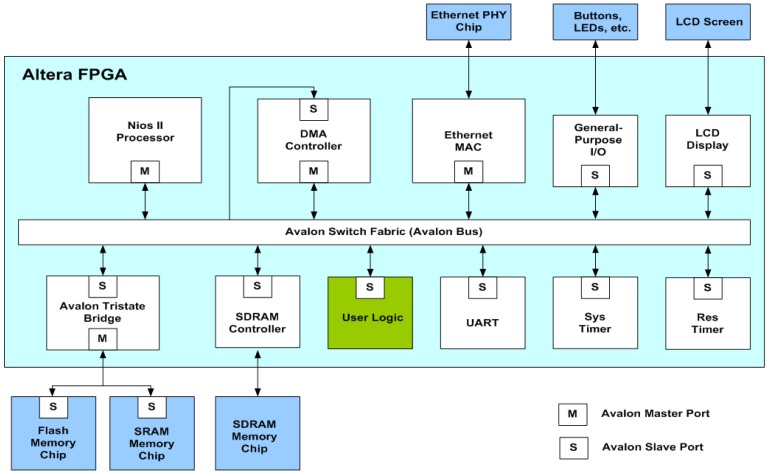
The SOPC architecture.

**Figure 16.**
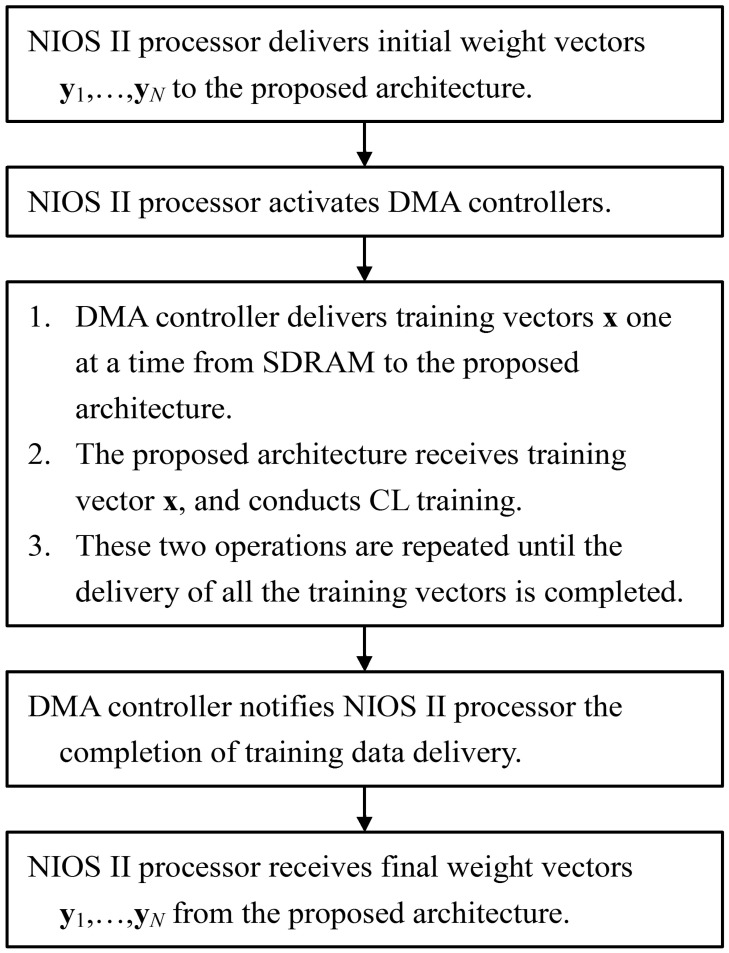
The flowchart of operations in the SOPC.

The proposed architecture can be employed in conjunction with the softcore processor for on-chip learning. As depicted in Figure 15, the proposed architecture is used as a custom user logic in a system-on-programmable-chip (SOPC) consisting of softcore NIOS II processor, DMA controller and SDRAM controller for controlling off-chip SDRAM memory. Figure 16 shows the operations of the SOPC for on-chip learning. From the flowchart shown in Figure 16, we see that the NIOS II processor is used only for the initialization of the proposed architecture and DMA controller, and the collection of the final training results. It does not participate in the CL training and data delivery. In fact, only the proposed architecture is responsible for CL training. The input vectors for the CL training are delivered by the DMA controller. In the SOPC system, the training vectors are stored in the SDRAM. Therefore, the DMA controller delivers training vectors from the SDRAM to the proposed architecture. After the CL training is completed, the NIOS II processor then retrieves the resulting neurons from the proposed architecture. All the operations are performed on a single FPGA chip. The on-chip learning is well-suited for applications requiring both high portability and fast computation.

## 3. Experimental Results

This section presents some numerical results of the proposed CL architecture. The design platform for the experiments is Altera Quartus II with SOPC Builder and NIOS II IDE. The target FPGA device for the hardware design is Altera Cyclone III EP3C120 [31]. The vector dimension of neurons is *w* = 2 × 2.

Table 3 shows the area costs of the proposed architecture for different number of stages *N* with various *k*. There are three different types of area cost considered in this experiment: number of logic elements (LEs), number of embedded memory bits, and the number of embedded multipliers. For example, given *N* = 128 and *k* = 4, the architecture consumes 12398 LEs, which is 87% of the LE capacity of the target FPGA device. It can be observed from the figure that the area costs grow linearly with *N*. Therefore, it can be effectively used for systems requiring large number of neurons *N*. The LE consumption also increases linearly with *k* for a fixed *N*. This is because the number of LEs of the swap unit at each stage grows with *k*. However, since the number of squared distance calculations is independent of *k*, the embedded multiplier consumption remains the same.

Performance analyses for different architectures are presented in Table 4. The area complexity of an architecture is the number of comparators and/or processing elements in the circuit. The latency represents the time taken by the architecture to finish one competition. The throughput means the number of competitions completed per unit of time. It can be deduced from the table that the proposed architecture achieves a balance between speed and space. The proposed architecture also can apply to applications which perform specific operations on *k* targets found within the input set. As compared to architectures in [12,32] and [33] for the *k*NN application, the proposed architecture takes advantage of the pipeline fashion to have higher throughput even though these architectures have same latency of picking out *k* winners.

The proposed architecture is adopted as a hardware accelerator of a NIOS II softcore processor. Tables 5 and 6 shows the CPU time of the proposed hardware architecture, its software counterpart and the hardware architecture proposed in [12] for various *k* and *N* values. The CPU time is the execution time of the processor for the CL over the entire training set. The software implementation is executed on the 2.8-GHz Pentium IV CPU with 1.5-Gbyte DDRII SDRAM. The architecture presented in [12] is also adopted as an accelerator for NIOS II softcore processor running on 50 MHz. The corresponding SOPC system is implemented in the same FPGA device Altera Cyclone III EP3C120. All implementations share the same set of training vectors with 65536 training vectors obtained from the 512 × 512 training image “Lena”.

We can see from Table 5 that the CPU time of the proposed architecture is lower than the other implementations. In fact, because of the pipeline implementation, the CPU time of the proposed architecture is almost independent of *N*. However, for the other implementations, the CPU time may grow linearly with *N*. Therefore, as *N* becomes large, the proposed architecture will have significantly higher speed for the CL training.

It can be noted from Table 6 that the CPU time increases with *k*. This is because the throughput of the proposed architecture decreases when *k* increases. Nevertheless, the speedup over its software counterpart is still high even for large *k* values.

To further illustrate the effectiveness of the proposed architecture, speedups of the proposed architecture over the software implementation and over the architecture presented in [12] are revealed in Table 7. It is not surprising to see that the speedup increases with *N*. In particular, when *N* = 128 and *k* = 4, the speedup over the software implementation attains 318.

In Table 8, we compare the proposed architecture with the architectures presented in [34,35] for clustering operations. The architectures in [34,35] are pipelined circuits for *c*-means and fuzzy *c* means algorithms, respectively. All the architectures have the same dimension *w* = 2 × 2. They all are used as hardware accelerators of the NIOS CPU for the computation time measurement. The area costs, computation time, and estimated power dissipation are considered in the comparisons. The power estimation is based on the PowerPlay Power Analyzer Tool provided by Altera. Note that direct comparisons of these architectures may be difficult because they are based on different algorithms. In addition, they are implemented on different FPGA devices. However, it can still be observed from the table that the proposed architecture has lower area costs as compared with the architectures in [34,35]. In addition, with larger training set and number of clusters, the architecture is able to perform the clustering with less computational time as compared with the architecture in [35]. The proposed architecture also has significantly lower power dissipation as compared with the architecture in [34]. The proposed architecture therefore has the advantages of low area costs, fast computation and low power consumption.

Table 9 reveals the dependence of the power consumption of the proposed architecture on the *k* and *N*. It can be observed from the table that the power dissipation of the proposed architecture only slightly grows as *k* and/or *N* increase. In particular, when *k* = 4, eight-fold increase in *N* (*i.e.*, from *N* = 16 to 128) leads to only 49.46% increase (*i.e.*, from 134.49 mW to 201.01 mW) in power consumption. Alternatively, when *N* = 128, four-fold increase in *k* (*i.e.*, from *k* = 1 to *k* = 4) results in only 14.47% increase (*i.e.*, from 175.59 mW to 201.01 mW) in power dissipation. Therefore, the proposed circuit is able to maintain lower power dissipation even for large *k* and *N* values.

An application for clustering operations is the image coding. The neurons obtained after CL training can be used as the codewords of a vector quantizer (VQ). Because there are *N* neurons in a CL network, the CL-based VQ contains *N* codewords. An image coding technique using VQ involves the mapping of each input image block **x** into a codeword. For a full-search VQ, the selected codeword is the closest codeword to **x**. Therefore, the average mean squared error (*MSE*) of the VQ is defined as

(6) $$\text{MSE}=\frac{1}{wt}\sum_{j=1}^{t}D({\text{x}}_{j},{\text{y}}_{\alpha (x_{j})})$$

where *w* is the vector dimension, *t* is the number of training vectors, and *α*() is the mapping given by

(7) $$\alpha (\text{x})=\arg\underset{1\leq i\leq N}{\min}D(\text{x},{\text{y}}_{i})$$

In addition to *MSE*, another commonly used performance measure is the peak SNR (*PSNR*), which is defined as

(8) $$\text{PSNR}=10\log\frac{255^{2}}{\text{MSE}}$$

Table 10 shows the performance of the proposed architecture for VQ with *N* = 64 and *w* = 2 × 2 for the two 512 × 512 test images “Houes” and “Lena.” The data set for CL training contains two 512 × 512 images “Baboo” and “Bridge.” The performance of software CL training is also included in the table for comparison purpose. It can be observed from table that only a small degradation in PSNR is observed for hardware implementation. The degradation mainly arises from the finite precision (*i.e.*, 8-bit) fixed-point number representations and the lookup table based division adopted by the hardware. Nevertheless, the degradation is small as observed in the table. All these facts demonstrate the effectiveness of the proposed architecture.

## 4. Concluding Remarks

A high-speed and area-efficient pipelined architecture for *k*WTA operations has been proposed. With the aid of codeword swapping scheme, the system throughput soared due to the ability to perform competitions associated with different training vectors in parallel. The CPU time of the architecture is almost independent of the number of neurons *N*, and the architecture is able to attain high speedup over other hardware or software implementations for large *N*. The hardware resources are effectively saved by only involving comparators and multiplexers in the *k*WTA operation. The utilization of lookup table based circuit for finite precision division further reduces the computational time and lowers the area cost in learning process. The proposed architecture has no extra cost on retrieving winners after identifying the targets, which is beneficial for those applications that operations only perform on small number of targets selected from a large input set. Our numerical results demonstrate these virtues of the proposed architecture.

## Figures and Tables

**Table 1.** The truth table for the multiplexer in the swap unit at stage *i* for *k* = 2.

***c_i_***	***c_i_*_+1_**	***c_i_*_+2_**	**MUX_out**	**Comments**
0	0	1	**y***_i_*_+2_	**x** is at stage *i* + 2. Stage *i* + 2 loses competition.
0	0	2	**y***_i_*_+1_	**x** is at stage *i* + 2. Stage *i* + 1 loses competition.
0	0	3	**y***_i_*	**x** is at stage *i* + 2. Stage *i* loses competition.
0	1	0	**y***_i_*	**x** is at stage *i* + 1. Stage *i* + 1 loses competition.
0	2	0	**y***_i_*_−1_	**x** is at stage *i* + 1. Stage *i* loses competition.
0	3	0	**y***_i_*	**x** is at stage *i* + 1. Stage *i* − 1 loses competition.
1	0	0	**y***_i_*_−2_	**x** is at stage *i*. Stage *i* loses competition.
2	0	0	**y***_i_*	**x** is at stage *i*. Stage *i* − 1 loses competition.
3	0	0	**y***_i_*	**x** is at stage *i*. Stage *i* − 2 loses competition.
0	0	0	**y***_i_*	Pipeline is in the initial state
Others			Error	Other combinations are errors.

**Table 2.** The updating process of *D*_*min*_1__, …, *D_min_k__* of the distribution unit at stage *i* for *k* = 2.

***c_i_***	**Neuron Failing the Competition**	**Updating Operation**	**Comments**
1	Stage *i*	New *D*_*min*1_ ← old *D_min_*_2_	Swap between stages *i* and *i* − 2
		New *D_min_*_2_ ← old *D*_*min*1_	
2	Stage *i* − 1	New *D*_*min*1_ ← *D_i_*	Swap between stages *i* − 1 and *i* − 2
		New *D_min_*_2_ ← old *D_min_*_2_	
3	Stage *i* − 2	New *D*_*min*1_ ← *D_i_*	No Swapping is necessary
		New *D_min_*_2_ ← old *D*_*min*1_	

**Table 3.** The area costs of the proposed architecture for different number of stages *N* with various *k*.

***N***	***k***	**LEs**	**Memory Bits**	**Embedded Multipliers**
	1	5320/119088 (4 %)	0/3981312 (0 %)	72/576 (13 %)
16	2	10353/119088 (9 %)	0/3981312 (0 %)	136/576 (24 %)
	3	20512/119088 (17 %)	0/3981312 (0 %)	264/576 (46 %)
	4	40449/119088 (34 %)	0/3981312 (0 %)	520/576 (90 %)

	1	8244/119088 (7 %)	0/3981312 (0 %)	80/576 (14 %)
32	2	15892/119088 (13 %)	0/3981312 (0 %)	144/576 (25 %)
	3	31236/119088 (26 %)	0/3981312 (0 %)	272/576 (47 %)
	4	62096/119088 (52 %)	0/3981312 (0 %)	528/576 (92 %)

	1	10754/119088 (9 %)	0/3981312 (0 %)	88/576 (15 %)
64	2	21297/119088 (18 %)	0/3981312 (0 %)	152/576 (26 %)
	3	42974/119088 (36 %)	0/3981312 (0 %)	280/576 (49 %)
	4	40449/119088 (72 %)	0/3981312 (0 %)	536/576 (93 %)

	1	12398/119088 (10 %)	0/3981312 (0 %)	96/576 (17 %)
128	2	25784/119088 (22 %)	0/3981312 (0 %)	160/576 (28 %)
	3	51729/119088 (43 %)	0/3981312 (0 %)	288/576 (50 %)
	4	104121/119088(87 %)	0/3981312 (0 %)	544/576 (94 %)

**Table 4.** Performance analyses for different architectures.

**Architecture**	[11]	[12]	[13]	[32]	[33]	**Proposed**
Area complexity	*O*(*N*^3^)	*O*(*N*)	*O*(*N*)	*O*(*N*)	*O*(*N*)	*O*(*N*)
Latency	*O*(1)	*O*(*N*)	*O*(*Nk*)	*O*(*N*)	*O*(*N*)	*O*(*N*)
Throughput	*O*(1)	*O*( $\frac{1}{N}$)	*O*( $\frac{1}{Nk}$)	*O*( $\frac{1}{N}$)	*O*( $\frac{m}{N}$)	*O*( $\frac{1}{k+1}$)
Pipeline	No	No	No	No	No	Yes
Comment				WTA only	*m*: # of modules	

**Table 5.** The CPU time of the proposed hardware architecture and the hardware architecture proposed in [12] for different *k* and *N* values (in *ms*).

	***k***							
	**1**		**2**		**3**		**4**	

*N*	Proposed Arch.	Arch. in [12]	Proposed Arch.	Arch. in [12]	Proposed Arch.	Arch. in [12]	Proposed Arch.	Arch. in [12]
16	7.52619	77.3898	7.5292	81.6892	7.52917	85.9886	7.52921	90.2881
32	7.52651	140.3564	7.53012	144.4846	7.52942	148.6127	7.52947	152.7408
64	7.52715	266.2679	7.90321	270.3023	7.90315	274.3367	7.5292	278.371
128	7.52843	518.1164	7.90309	522.1019	7.529	526.0874	7.90332	530.0729

**Table 6.** The CPU time of the proposed hardware architecture and its software counterpart for different *k* and *N* values. (in *ms*)

	***k***							
	**1**		**2**		**3**		**4**	

*N*	Proposed Arch.	Software	Proposed Arch.	Software	Proposed Arch.	Software	Proposed Arch.	Software
16	7.52619	300.75	7.5292	325.70	7.52917	353.439	7.52921	377.1
32	7.52651	585.03	7.53012	614.37	7.52942	651.777	7.52947	686.23
64	7.52715	1176.22	7.90321	1190.58	7.90315	1249.32	7.5292	1308.22
128	7.52843	2340.11	7.90309	2333.72	7.529	2427.26	7.90332	2511.97

**Table 7.** Speedups of the proposed architecture over its software counterpart and the architecture in [12] for different number of neurons *N* with various *k*.

	***k***							
	**1**		**2**		**3**		**4**	

*N*	Arch. in [12]	Software	Arch. in [12]	Software	Arch. in [12]	Software	Arch. in [12]	Software
16	10.2827	39.9605	10.8496	43.2582	11.4268	46.9426	11.9917	50.0849
32	18.6483	77.7293	19.1876	81.5766	19.7276	86.5640	20.2857	91.1392
64	35.3741	150.2637	34.2016	150.6451	34.7123	158.0787	36.9722	173.7529
128	68.8213	310.8364	66.0630	295.2921	69.8748	322.3881	67.0696	317.8373

**Table 8.** Comparisons of various architectures in for clustering applications for dimension *w* = 2 × 2.

	**Proposed Architecture** **(*k* = 2, *N* = 64)**	**Architecture in** [34] **(*N* = 64)**	**Architecture in** [35] **(*N* = 16)**
FPGA	Altera	Altera	Altera
Devices Size of	Cyclone III EP3C120	Stratix II EP2S60	Cyclone III EP3C120
Training Set LEs/ Adaptive	65,536	64,000	60,000
Logic Modules	21,297	13,225	51,832
(ALMs) Embedded	LEs	ALMs	LEs
Multipliers/	152	288	288
DSP Blocks Embedded	Multipliers	DSP Blocks	Multipliers
Memory Bits Computation	0	8192	312,320
Time Estimated	7.90 ms	7.04 ms	22.86 ms
Power Consumption	155.50 mW	860.08 mW	137.45 mW

**Table 9.** Power consumption estimation of the proposed architecture (mW) for various *k* and *N* values.

	***k***			
***N***	**1**	**2**	**3**	**4**
16	131.07	131.77	132.81	134.49
32	137.86	138.96	141.32	145.45
64	149.84	155.50	161.84	168.87
128	175.59	185.05	193.54	201.01

**Table 10.** Performance of the proposed architecture for image compression for *N* = 64 and dimensions *w* = 2 × 2.

**Images**		**House**	**Lena**
Software	*MSE*	150.0826	242.5662
	*PSNR*	26.3675 (dB)	24.2825 (dB)
Proposed Arch.	*MSE*	150.1482	242.6272
	*PSNR*	26.3656 (dB)	24.2814 (dB)
